# Proceedings of the Oncofertility Congress of the “Freezing Ovarian Tissue and Oocytes” (FOTO) Consortium Brussels

**DOI:** 10.1007/s10815-022-02552-7

**Published:** 2022-06-25

**Authors:** Marie-Madeleine Dolmans, Isabelle Demeestere, Ellen Anckaert, Michel De Vos

**Affiliations:** 1 Gynecology Department, Cliniques Universitaires St-Luc, Avenue Hippocrate 10, 1200 Brussels, Belgium; 2 Gynecology Research Laboratory, Institut de Recherche Expérimentale Et Clinique, Université Catholique de Louvain, Avenue Mounier 52, bte B1.52.02, 1200 Brussels, Belgium; 3 Research Laboratory On Human Reproduction, Fertility Clinic, CUB-Hôpital Erasme, Université Libre de Bruxelles (ULB), Brussels, Belgium; 4 Follicle Biology Laboratory, Vrije Universiteit Brussel (VUB), 1090 Brussels, Belgium; 5 Brussels IVF, Universitair Ziekenhuis Brussel, Vrije Universiteit Brussel, Laarbeeklaan 101, 1090 Brussels, Belgium

Since the nascence of oncofertility, a discipline striving to mitigate the impact from cancer and cancer treatment on fertility, multicenter networks have been set up to share knowledge, enhance clinical expertise in referral centers, and facilitate follow-up studies of patient cohorts. In 2018, three pioneering centers in fertility preservation located in the Brussels Capital Region in Belgium joined forces to establish an oncofertility research consortium named “Freezing Ovarian Tissue and Oocytes (FOTO) Consortium” with the aim of strengthening multicenter scientific collaboration. This consortium consolidates the existing clinical collaboration between these centers which led to the first birth of a healthy child from transplanted frozen-thawed ovarian tissue in 2004 [1].

Four years of fundamental research funded by an Excellence of Science (EOS) grant by FWO-FNRS have culminated in a 2-day scientific oncofertility conference in October 2021 featuring the latest developments and advances in the field. Trending keynote topics at this virtual conference included acquisition of oocyte competence and updated insights into mechanisms of chemotherapy-induced ovarian follicular loss. Presentations of the latest results of basic and translational research on female fertility preservation within the consortium alternated with an overview of current clinical options of fertility preservation in girls and women with cancer.

## Acquisition of oocyte competence: from the primordial to pre-ovulatory stages

David Albertini (USA) summarized the many years of research into the oocyte’s journey towards a state conducive to fertilization, embryo development, and subsequent pregnancy. In order for the oocyte to contribute to the establishment and maintenance of a term pregnancy, it must acquire and execute a strict sequence of processes critical to the completion of oogenesis and early embryogenesis. Referred to as developmental competencies, the female gamete acquires the ability to complete meiosis, engage in and effect fertilization, and sustain preimplantation embryonic development at sequential stages of its intrafollicular differentiation. His lecture highlighted the role of oocyte-specific gene products known to regulate three critical stages of development in mammals. The transition in the cell cycle from that of meiosis to mitosis following fertilization is manifest by the expression and subsequent degradation of proteins that drive meiotic maturation during ovulation and ensure entry into embryonic interphase. Next, the competence to shift gene expression from that of the oocyte to the embryo, so-called embryonic gene activation or EGA, was discussed in the context of epigenetic modifications to the genome that eventually enable development up to the time of implantation. The competence to effect and direct blastomere polarization of the compacting embryo was then discussed in the context of maternal factors, such as the subcortical maternal complex and actin cytoskeleton, through the assignment of inner and outer lineages that specify the inner cell mass and trophectoderm. The lecture concluded by emphasizing that most of our knowledge regarding oocyte competencies has been derived from studies on mouse embryos and given the high incidence of embryonic arrest in human IVF, there is a string need to understand the problem of oocyte competency acquisition in humans, especially as it pertains to the growing field of fertility preservation.

## Ovarian tissue cryopreservation and transplantation

Researchers from Marie-Madeleine Dolmans’ team (Belgium) presented their lab results in the field of ovarian tissue transplantation (OTT), focusing on improvement of follicle survival after OTT and strategies to avoid malignant cell reseeding. Indeed, the effectiveness of OTT is limited by the massive follicle loss, reaching up to 90%, which occurs in the early post-grafting period due to hypoxia. Graft revascularization occurs progressively through bidirectional neoangiogenesis, resulting in reoxygenation kinetics characterized by oxidative stress around 10 days after transplantation [2].

Numerous strategies have been developed to reduce ovarian post-grafting injury and improve follicle outcomes. These include the addition of proangiogenic growth factors, hormones, and various antioxidants which have shown to boost revascularization or reduce oxidative stress, although there is little evidence of a positive impact on follicle survival. Luciana Cacciottola (Belgium) addressed the use of adipose tissue-derived stem cells in the context of OTT and showed favorable data of reduced hypoxia, enhanced revascularization, and primordial follicle quiescence [3].

One overriding safety concern when considering OTT in patients in remission after cancer is the risk of (minimal) residual disease (MRD) in cryopreserved ovarian cortex. Malignant cells have been detected in patients with acute leukemia [4–7]. Although OTT is considered safe in several other diseases (for review, see [8, 9]), data on the safety of OTT in patients suffering from central nervous system (CNS) tumors have been lacking. Thi Yen Thu Nguyen initiated a study with the aim of investigating the possible presence of cancer cells in cryopreserved and xenografted ovarian tissue from 21 patients with CNS tumors. Long-term (5 months) xenografting to immunodeficient mice was performed. The presence of malignant cells was investigated with disease-specific markers in each patient’s cryopreserved and xenografted ovarian tissue by histology, immunohistochemistry (IHC) via expression of neuron-specific enolase (NSE) and glial fibrillary acidic protein (GFAP), and reverse transcription droplet digital polymerase chain reaction (RT-ddPCR) for quantification of GFAP and ENO2 gene amplification. No malignant cells were detected by histology in frozen-thawed and xenografted ovarian tissue from any of the 21 patients. All samples were negative for NSE and GFAP, although these neural markers were expressed extensively in the patients’ primary tumors. Likewise, no malignant involvement was detected in cryopreserved and xenografted ovarian fragments from subjects with CNS tumors using RT-ddPCR. In summary, OTT in patients with CNS tumors can be considered safe, as there was no evidence of malignant cell seeding in frozen-thawed and xenotransplanted ovarian tissue from these patients [10–12]. Nevertheless, a case-by-case evaluation according to tumor type and specific markers expressed by the primary tumor remains required. To increase the sensitivity of malignant cell detection, a combination of available approaches including histology, IHC, molecular biology, and xenotransplantation is mandatory.

Camille Hossay (Belgium) presented her study designed to elucidate whether ovarian tissue is able to withstand a double freezing–thawing procedure. This innovative approach should be suitable for patients who had a whole ovary cryopreserved and should allow for a safe transplantation of their frozen tissue using the following procedure: 1/ thawing the whole ovary; 2/ dissecting the ovary into cortical strips; 3/ selecting fragments for MRD screening; and 4/ refreezing the resulting strips. Moreover, other indications for refreezing would be to avoid wastage of ovarian tissue, which is very precious, and to promote collaboration between centers in order to accomplish large-scale and conclusive studies in the field. To do so, human ovarian cortical biopsies from four thawed whole ovaries were divided into four experimental subgroups: (a) a frozen-thawed, non-grafted group; (b) a frozen-thawed, xenografted group; (c) a refrozen-rethawed, non-grafted group; and (d) a refrozen-rethawed, xenografted group. Xenografting was performed using eight severely combined-immunodeficient mice for a total duration of 21 days. Follicle density, classification, growth, atresia rate, and ultrastructure were studied, as well as tissue fibrosis and vascularization. Morphologically normal preantral follicles were detected in all groups. Camille Hossay observed a dramatic decline of more than 65% in the proportion of early preantral follicles after grafting of frozen-thawed and refrozen-rethawed ovarian tissue. Moreover, mean follicle densities remained comparable between the frozen-thawed and refrozen-rethawed tissues, both in the non-grafted and grafted groups. Equivalent proportions of proliferating early preantral follicles were identified in frozen-thawed and refrozen-rethawed samples, whether the tissue was grafted or not. Furthermore, she did not observe any significant differences in atretic follicle rates between any of the four groups, and the ultrastructural quality of follicles appeared unaffected by the refreezing procedure. Similar proportions of fibrosis were noted in the frozen-thawed and refrozen-rethawed groups, irrespective of grafting. Finally, no significant differences were noted in terms of vascularization when comparing the two freezing conditions. These observations led her to suggest that refrozen-rethawed ovarian tissue retains the same functional characteristics as frozen-thawed ovarian tissue [13].

## Follicular activation in ovarian tissue

### Follicular activation in frozen-thawed and grafted human ovarian tissue

Camille Hossay (Belgium) went on to present an ongoing project to elucidate the mechanisms by which follicles activate after OTT, resulting in massive follicle loss in the ovarian graft. She focused on the role of specific proteins involved in the PI3K (Akt, mTOR, and FOXO1) and Hippo (YAP) signaling pathways. Frozen-thawed ovarian tissue was collected from six patients and divided into four fragments for each patient (one control fragment and three fragments grafted to immunodeficient mice for 3, 7, and 21 days). Activation of the PI3K and Hippo signaling pathways was investigated by analysis of Akt and mTOR phosphorylation, FOXO1 cytoplasmic localization, and YAP nuclear localization. A significant decrease in primordial follicle density associated with a significant increase in growing follicle density was detected as soon as 3 days after transplantation. However, tissue grafting also induced follicular death with more than 50% of follicles being atretic after 3 days, and a further 50% after 7 days of grafting [14]. As far as follicular activation through the PI3K pathway is concerned, Akt phosphorylation levels were significantly elevated in primordial follicles after 3 days of grafting, reflecting its role in the follicular activation process. Furthermore, a higher percentage of primordial follicles with cytoplasmic FOXO1 was observed after grafting, confirming the Akt-mediated role in follicular activation. However, mTOR was suggested to be involved in sustaining further follicular growth rather than in the onset of primordial follicular activation. Finally, a greater proportion of primordial follicles was detected with nuclear YAP at all timepoints after grafting, suggesting a Hippo-mediated action in follicular activation. While documenting the involvement of the PI3K and Hippo pathway effectors in follicle burnout after OTT, this study supports the hypothesis that follicular loss occurs as an early event after transplantation, with follicular growth and death both contributing to the burnout phenomenon [15].

### Follicular activation in cultured ovarian tissue

**Fig. 1:**
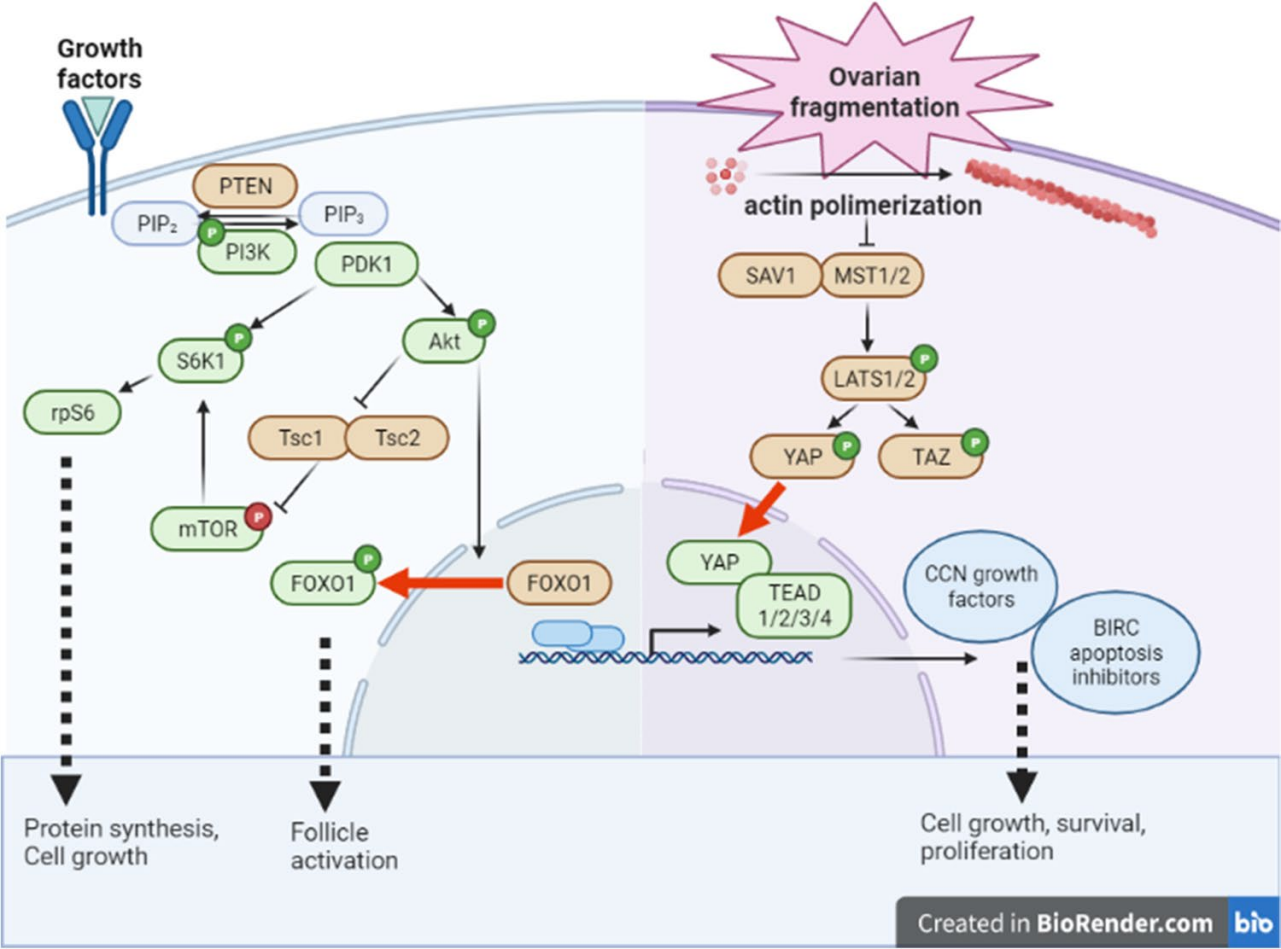
Representation of the PI3K and Hippo pathways involved in follicle activation. The PI3K pathway (left) is a phosphorylation cascade triggered by the binding of numerous cytokines and growth factors to tyrosine-kinase receptors on cell membranes. Upon Akt phosphorylation or inhibition of PTEN, a number of effectors are activated, including FOXO1 and mTOR, in order to initiate granulosa cell growth from flattened to cuboidal and early follicle growth. The Hippo pathway (right) can be disrupted by conversion of globular-actin into filamentous-actin. It works through its action on YAP/TAZ, which, once unphosphorylated, shifts to the nucleus to activate transcription factors like TEAD1-4, which are related to follicle activation and growth. Activators are represented in green; inhibitors are represented in orange. Abbreviations: Akt, protein kinase B; BIRC, baculoviral inhibitors of apoptosis; FOXO1, forkhead box O1; LATS1/2, large tumor suppressor kinase 1/2; mTOR, mechanistic target of rapamycin; MST1/2, mammalian Ste20-like serine/threonine kinases 1/2; PDK1, pyruvate dehydrogenase lipoamide kinase isozyme 1; PI3K, phosphoinositol-3-kinase; PIP2, phosphatidylinositol 4,5-bisphosphate; PIP3, phosphatidylinositol (3,4,5)-trisphosphate; PTEN, phosphatase and tensin homolog deleted on chromosome 10; rpS6, ribosomal protein S6; SAV1, protein Salvador homolog 1; S6K1, ribosomal protein S6 kinase beta-1; TAZ, transcriptional coactivator with PDZ-binding motif; TEAD 1/2/3/4, TEA domain family members 1/2/3/4; TSC1 and TSC2, tuberous sclerosis proteins 1 and 2; YAP, yes-associated protein. Adapted with permission from Dolmans MM, Donnez J, Cacciottola L. Trends Mol Med. 2021; 70:777–791

Researchers from Isabelle Demeestere’s team (Belgium) addressed the crucial role of follicular activation in the regulation of the ovarian reserve during physiological and non-physiological conditions using animal and human models (presented by C. Janssen, M. Devos and N. Donfack). The precise mechanisms by which the PI3K/AKT/mTOR and Hippo pathways are involved in the activation of primordial follicles remain poorly understood. Casper Jansen (Belgium) summarized his study to enhance our understanding of the interaction of these two pathways and the impact of tissue processing and-chemotherapy on follicle activation using a postnatal day 3 (PND3) mouse model, containing quiescent follicles at primordial and primary stages. The involvement of the PI3K/Akt/mTOR and Hippo signaling pathways in in vitro spontaneous and induced follicular activation conditions was evaluated after tissue fragmentation (depicted in Fig. 1) and after chemotherapy exposure. Intact and fragmented PND3 mouse ovaries were cultured for up to 48 h in control medium (spontaneous activation group) or exposed to a cyclophosphamide metabolite (4-hydroperoxycyclophosphamide (4HC), 3 µM or 20 µM for 4 h or 24 h-chemotherapy-induced follicular activation) [16]. In the in vitro spontaneous activation condition, p-Akt levels rapidly decreased as compared to uncultured ovaries, while PI3K/Akt/mTOR pathway gene expression levels were not altered. The drop in the expression of the phosphorylated form of PI3K/Akt/mTOR effectors could be explained by the absence of growth factors in the medium capable of activating this pathway. In contrast, the expression levels of *ccn2* and *cMyc*, two genes regulated by the Hippo pathway, were increased after 4 h of culture, even when ovaries were cultured as intact organs, suggesting that the removal of the ovaries from their physiological environment is sufficient to induce non-physiological follicular activation through the Hippo pathway. Consequently, the proportion of growing follicles significantly increased after 2 days. At the dose used in this experiment, chemotherapy exposure (4HC) did not inhibit follicular growth but induced a dose-dependent increase in apoptosis and morphological abnormalities. In the presence of 4HC, the phosphorylation levels of PI3K pathway proteins, such as Akt and rpS6, were increased at 4 and 48 h of culture as well as the *cnn2* gene expression level at 48 h of culture. These data highlight the major involvement of both PI3K/Akt/mTOR and Hippo pathways in in vitro spontaneous and induced follicle activation [16]. Melody Devos (Belgium) studied the impact of chemotherapy on follicles prior to ovarian tissue cryopreservation was assessed in quiescent follicles of adult and prepubertal patients using tissue donated for research at the end of the storage period. After 24 h of culture, the proportion of follicles with DNA damage was higher in cortex exposed to chemotherapy compared to non-exposed cortex (unpublished data). Analysis of the signaling pathways after thawing showed a higher expression of phosphorylated Akt and rpS6 in chemotherapy exposed cortex compared to unexposed cortex, irrespective of age. Immunostaining localized pAkt in the oocyte while p-rpS6 was more pronounced in granulosa cells, suggesting an early process of the follicle activation through the PI3K pathway. These preliminary data confirm the impact of previous chemotherapy exposure on follicular activation and survival in human ovarian cortex.

The team of Isabelle Demeestere further hypothesized that biochemical inhibition of the PI3K/Akt pathway could limit spontaneous follicular activation and improve the in vitro growth process. To investigate this, the team used Everolimus (EVE), a rapamycin analog that acts on the mammalian target of rapamycin (mTORC1); EVE has been used for its potent antiproliferative effects in human endocrine tumors and for improving organ function after transplantation. Using a mouse model, the team observed significantly decreased P-rps6 levels, without affecting P-Akt levels, confirming that EVE was effective to inhibit mTORC1. Surprisingly, EVE was also able to prevent an increase in CCN2 expression after 4HC exposure at 48 h of culture, suggesting a cross-talk between both signaling pathways [16]. Melody Devos further elaborated on the hypothesis that EVE might slow down the initiation of human primordial follicle growth, allowing follicles to develop within a protracted timeframe, more closely resembling physiological conditions [17, 18]. Using human ovarian cortex donated for research, she showed that PI3K/Akt inhibitors partially safeguard the follicular reserve at the earliest stages while PI3K activators seemed to accelerate the initiation of in vitro follicular growth with an increased switch from the primordial to transitory stage. In contrast, Hippo pathway disruption, through ovarian fragmentation, reversed the potential benefit of EVE on human growing follicles after 6 days of culture. In vitro generated follicles expressed markers of healthy follicular development but presented structural abnormalities. These observations raise additional questions on the potential interaction between both pathways in the ovary [19].

On the other hand, Nathalie Donfack (Belgium) presented data on the effectiveness of Hippo pathway inhibitors to reduce the extent of follicular activation as a result of tissue processing and culture. Mouse ovaries and bovine ovarian cortex were exposed in vitro to Verteporfin, a pharmacological inhibitor of YAP (unpublished data). In both models, Verteporfin had a moderate effect on the Hippo pathways effector with a dose-dependent increasing of toxicities. Although Verteporfin partially blocked follicular activation after fragmentation, follicular activation induced by chemotherapy exposure was not reduced. Altogether, these results highlighted the complexity of the regulation of follicular activation in non-physiological conditions and point towards the importance of a multitargeted approach with a combination of drugs to control induced dysregulation of in vitro follicle growth, or during ovarian processing for fertility preservation [19].

The second topic elaborated by the ULB team was the development of an innovative pharmacoprotective approach to prevent chemotherapy-induced ovarian damage and was presented by Chrisanthi Alexandri and An Nguyen (Belgium). According to recent studies, the miRNA technology has emerged as a promising fertoprotective tool [20]. In the ovary, miRNAs have been involved in follicle development as regulators of follicular growth, atresia, and steroidogenesis [20]. miRNAs are also involved in DNA damage repair and can be modulated themselves during chemotherapy. Chrisanthi Alexandri previously demonstrated that miRNAs are differentially expressed in the ovary exposed to chemotherapy and identified miRNAs involved in gonadotoxicity [21, 22]. Among the up/down-regulated miRNAs identified by TaqMan Low Density Arrays, 21 miRNAs with a potentially important biological function were selected for further individual validation. Among those, miRNA let-7A was most dysregulated while the in silico target identification and pathway enrichment analysis showed that its targeted genes are implicated in important biological processes. After in vitro transfection of mimic-miRNAs (let-7A) as replacement therapy, Chrisanthi Alexandri showed that chemotherapy-ovarian damage can be reduced in a mouse model [21]. She then transfected PND3 ovaries in vitro using lipofectamine; she exposed these ovaries to cyclophosphamide metabolites before being grafted into the kidney capsule of adult female mice in order to assess the follicular growth and ability of the oocytes to acquire maturation competence and used non-exposed ovaries as controls (unpublished data). In vivo follicular development after 3 weeks showed that let-7a restoration during chemotherapy exposure reduced apoptosis and tended to slow down follicle activation. The in vitro oocyte maturation rate was higher when let-7a was restored after chemotherapy exposure compared to chemotherapy exposure alone, suggesting a potential long-term benefit of this approach on oocyte quality. However, to apply this technology in vivo and target the ovary, a new delivery system is currently being developed using “organ-targeted” functionalized Gold nanoparticles, selected for their capacity to become functionalized, their bio-compatibility and immunogenicity. The preliminary results of this approach in cell lines and in vitro grown follicles were presented by An Nguyen (Belgium). She confirmed the low cytotoxicity of these Gold nanoparticles, the efficiency of their uptake in cells, and their intracellular stability (unpublished data). These results offer a new insight in the attractive pharmacological options to preserve fertility not only in women but also potentially in men in the future [23].

## In vitro maturation of oocytes

Finally, researchers from the team of Ellen Anckaert (Belgium) presented their lab results in the field of oocyte in vitro maturation (IVM) and in vitro follicle culture (IFC).

When there is a risk for reintroducing malignant cells, transplantation of ovarian cortical tissue is contraindicated. In these patients, efficient in vitro culture systems to support follicle growth and maturation from the earliest stages may be a future option for preserving fertility. Anamaria-Cristina Herta (Belgium) presented her work in a properly characterized in vitro follicle culture system in the mouse model [24]. In this system, following mechanical isolation from prepubertal mice ovaries, secondary follicles have been cultured individually during 10 days in a two-dimensional (2D) attachment system, generating fertilizable mature oocytes at high rates. However, the developmental competence of the cultured oocytes requires further improvement. To identify deviations from the situation in vivo, Anamaria-Cristina Herta performed a baseline carbohydrate metabolism characterization of in vitro and in vivo grown and matured mouse antral follicles both at the immature (GV) and mature (MII) stages. Targets for glucose related pathways were measured by direct enzymatic assays (with spectrophotometric detection) and gene expression (qRT-PCR) analysis [25] in the oocyte and somatic cell compartments of the follicle. This study revealed that all cell types from in vivo antral follicles exhibit glycolysis, citric acid cycle, and pentose phosphate pathway (PPP) activity. At the GV stage, in vivo antral follicle granulosa cells have higher lactic acid fermentation compared to cumulus cells, while oocytes present more PPP activity. Overall, increased metabolic activity in the different pathways was detected in granulosa cells compared to cumulus cells at the GV stage, even though the level of significance was not always reached. Ovulation triggering boosts pyruvate and lactate uptake, leads to consumption of reduced nicotinamide adenine dinucleotide phosphate (NADPH) generating the oxidized form (NADP +), and increases small molecules antioxidant capacity (SMAC) and TCA cycle activities in CCs in vivo. In contrast, the metabolic upregulation triggered by ovulation in the aforementioned pathways in CCs is limited in vitro. These alterations might be due to cell exhaustion due to the in vitro culture conditions, impairing cumulus cells in providing proper metabolic support to the oocyte. The observed deviations in metabolic pathways in vitro are a target for future culture optimization strategies.

The mouse IFC model may also serve as a tool to develop successful strategies to rescue human oocyte competence in the context of biphasic IVM, i.e., IVM preceded by a capacitation culture step (CAPA-IVM) in which maintenance of the oocyte-somatic connections is important [26]. Following the retrieval of COCs from small or medium-sized antral follicles for IVM, a proportion of the COCs may be partially denuded. These oocytes can mature following IVM, but their developmental competence remains low (unpublished data). Anamaria-Cristina Herta aimed to use the mouse IFC model to assess the oocyte’s ability to reconnect with its somatic companions in vitro, hypothesizing that the developmental competence of these oocytes could be rescued by the reconnection. She performed mouse IFC for 10 days. On culture day 5, when follicles were either at the preantral or early antral stage [27], in a proportion of the follicles, the oocyte was completely mechanically denuded. After denuding, the oocytes were put back in culture for 5 days, on top of their somatic cells, while the remainder of follicles were left intact. On culture day 9, the oocyte-somatic reconnection rate, the presence of transzonal projections (TZPs), and oocyte growth were assessed; on day 10, after hCG/EGF stimulation, oocyte maturation rate was assessed and developmental competence was studied by in vitro fertilization (IVF) and gene expression analysis. The results of the study indicated that in vitro grown oocytes can reconnect with their somatic companions if isolated at preantral and early antral stages, with no impact on follicle morphology and oocyte competence [28]. These novel findings constitute good premises for developing successful strategies to rescue human oocyte competence in the context of biphasic IVM.

Nazli Akin (Belgium) discussed the ongoing research to optimize a biphasic IVM system named CAPA-IVM as a tool for oncofertility preservation. She reviewed the use of the oocyte meiotic inhibitor C-natriuretic peptide (CNP) in the pre-maturation phase of the biphasic IVM system, before resumption of meiosis is induced by EGF-like peptides and FSH in the IVM phase. Oocyte secreted factors (OSFs) GDF9 and BMP15 are important regulators of CC function and have been shown to enhance oocyte quality and subsequent embryo development [29]. Both proteins consist of pro- and mature-domains and they are present in homo- and heterodimeric forms. Prodomains mediate the folding and the dimerization of OSFs. The heterodimer Pro-Cumulin activates SMAD2/3 and SMAD 1/5/8, and its efficiency in IVM systems over mature Cumulin and homodimeric OSF forms has been documented in animal models [30]. Stocker et al. have engineered a modified version of GDF9, named Super-GDF9, containing BMP15 finger residues in the receptor binding region, which is more than 1000-fold more bioactive than wildtype GDF9 and which potently activates the SMAD2/3 pathway [31]. Nazli Akin has tested both Pro-Cumulin and Super-GDF9 in the mouse CAPA-IVM system. Juvenile unprimed mice of 19 to 21 days of age were used, to serve as a model for oocytes with low developmental competence, comparable to human IVM performed with COCs from small antral follicles. Nazli Akin added the factors either only in the pre-IVM (for Pro-Cumulin) or both pre-IVM and IVM phases (for both OSFs). According to her results, oocyte competence remained unchanged following culture with OSFs as assessed by embryologic outcomes and total number of blastomeres in day 5 blastocysts. However, culturing with OSFs resulted in larger, more mucified COCs, and this observation was also reflected in CC gene expression of ovulatory cascade genes, which was more comparable to the in vivo situation for CAPA-IVM in the presence of OSFs [32]. Since embryological outcomes in human CAPA-IVM were less favorable than in the mouse model, and in view of the positive effects of OSFs on mouse CC function, studies on the effect of OSFs in human biphasic IVM are warranted.

Berta Cava Cami (Belgium) assessed the effect of Pro-Cumulin in addition to human COCs matured in vitro using the biphasic CAPA-IVM system. COCs were retrieved from antral follicles with a diameter of < 9 mm in dysovulatory patients with polycystic ovary syndrome (PCOS) who had not received an ovulation trigger before oocyte retrieval. Sibling COCs were randomly allocated to the interventional and control arms to minimize the effect of patient-related variables. COCs were collected after the CAPA step at the GV stage and after the maturation step at the MII stage. Berta Cava Cami found that oocyte maturation rates were unaffected by the addition of Pro-Cumulin, whereas the proportion of apoptotic cumulus cells per COC at the GV stage was reduced. Her observations indicate that Pro-Cumulin supplementation promotes NSN-to-SN chromatin configuration transition in oocytes after the pre-maturation phase. Furthermore, based on her data, she suggested that supplementation of Pro-Cumulin might promote mitochondrial activity as assessed by JC-1 staining in mature oocytes. Further studies are warranted to assess the effect of Pro-Cumulin addition on oocyte developmental competence in human biphasic IVM.

Given the essential role of carbohydrate metabolism in acquisition of oocyte competency, Nazli Akin (Belgium) focused on the characterization of glucose metabolic pathways in COCs during CAPA-IVM. Using the same mouse model, glucose metabolism pathways were assessed (using qRT-PCR and enzymatic assays) in oocytes and cumulus cells from immature (GV, after pre-IVM) and mature (MII, after IVM) COCs [25] and compared to in vivo grown and matured counterparts. Her results confirmed the individual tasks of oocytes and cumulus cells in deviating glucose to pentose phosphate pathway and performing glycolysis and citric acid cycle (CAC), respectively. Furthermore, while in vivo CC glycolysis and CAC increased from the GV to MII stage, both pathways were already highly active in cumulus cells at the end of the pre-IVM step in CAPA-IVM, but the activity decreased during final maturation in vitro. This indicates that while CAPA-IVM cumulus cells can utilize glucose, their ability to support oocytes during final maturation is impaired. Future CAPA-IVM optimization strategies should focus on adjusting culture media energy substrate concentrations or adding OSFs which may enhance the metabolic function of cumulus cells.

Finally, Laura Saucedo-Cuevas (Belgium) presented her work performed in collaboration with Dr. Gavin Kelsey, Epigenetics Programme, Babraham Institute, Cambridge, UK, and focused on epigenetic safety of oocyte culture. There is concern that superovulation or oocyte/follicle culture might affect the timely acquisition of correct imprinting patterns in oocytes and the maintenance of genomic imprinting after fertilization. Until a few years ago, conventional bisulfite sequencing was used to assess the DNA methylation and only a limited number of imprinted genes were studied. Lately, DNA methylation was evaluated in MII oocytes obtained from adult and prepubertal mice by ovarian superovulation, following IFC from the early preantral stage and by natural ovulation by applying whole genome bisulfite sequencing (WGBS, [33]). Regardless of the treatment to mature oocyte or the sexual maturity of the animals, the global DNA methylation pattern was largely conserved. However, specific and recurrent differences in DNA methylation were found. Laura Saucedo-Cuevas explained that she expanded these previous findings by performing WGBS analysis of individual mouse blastocysts. By doing so, she provided a comprehensive analysis of the DNA methylation profile of blastocysts derived from IFC, from superovulated mice, and from non-stimulated mice. She showed that both hormone stimulation and sexual maturity are associated with very limited alterations in methylation of specific CpG islands and imprinted genes. More specifically, superovulation in adult mice was associated with alterations at the *Sgce* and *Zfp777* imprinted genes. DNA methylation at the *Sgce* germline differentially methylated region (gDMRs) was significantly reduced in the superovulation adult condition compared to natural ovulation while *Zfp777* gDMR showed higher methylation levels in the superovulation adult group. IFC-derived blastocysts showed a decrease in global methylation levels and increased variability in imprinted DNA methylation. Overall, this study serves as a baseline to design markers for ART protocol optimization studies.

## Fertility preservation research from bench to bedside

The second day of the meeting’s agenda was designed to achieve three goals: discuss the status of the clinical management of fertility preservation in cancer patients, assess the recent developments related to reproductive options in cancer survivors, and engage the speakers in a lively debate on how they would approach fertility preservation in specific clinical situations.

Dr Daniela Nogueira (INOVIE Fertilité, France) reviewed the establishment of networks for fertility preservation based on existing guidelines within Europe and from a local perspective in France. She addressed the recommendations of the European Society of Human Reproduction on how to organize the care for women undergoing fertility preservation within a single multidisciplinary clinical team and went on to review the structure of the “Onco Occitanie” Network, focusing on (1) the importance of multidisciplinary weekly meetings to facilitate the selection of optimal patient-tailored fertility preservation strategies, (2) readily available ovarian reserve testing, (3) linked databases for sharing medical records, and (4) efficient systems of tissue transport.

Professor Isabelle Demeestere (Belgium) reviewed the current status of ovarian stimulation (OS) with letrozole cotreatment and the use of the progestins instead of GnRH antagonists to suppress the LH peak. She emphasized that more than half of the patients who are confronting cancer at a young age are concerned about the impact of cancer on fertility. She reviewed the pros and cons of cryopreserving ovarian tissue and oocytes/embryos, which are the main options for female fertility preservation. Further to a concise overview of the modalities of ovarian stimulation, with a focus on the overall adoption of the random start OS, Demeestere summarized the live birth rates with vitrified oocytes according to the largest dataset available so far from the group of Cobo in Spain [34], pointing out to the increasing number of vitrified oocytes needed to obtain a live birth with advancing age. In drawing attention to the need to develop optimized OS protocols with minimal burden for the cancer patient, the available evidence regarding the use of the progesterone primed ovarian stimulation (PPOS) protocol in the fertility clinic was discussed, with a higher number of oocytes obtained after PPOS compared to standard OS according to a recent meta-analysis encompassing 9 studies and more than 1800 IVF patients [35]. Demeestere went on to present her experience with oocyte vitrification for fertility preservation in breast cancer patients, who account for 90% of all fertility preservation indications for cancer in her center. After referring to the original OS protocol with letrozole cotreatment (LET-OS) in breast cancer patients as developed by the group of Oktay et al. [36], she presented the data of the BROVALE study that was conducted in her center, comparing the safety and efficiency of LET-OS with that of matched controls from a standard IVF program, pointing towards increased serum progesterone (P) levels after oocyte retrieval in a LET-OS protocol initially proposed by Oktay et al. In view of uncertainties regarding the safety of elevated P levels in breast cancer patients, she suggested to omit letrozole after oocyte retrieval and to administer GnRH antagonists to enhance luteolysis and to lower serum P levels. As far as safety of LET-OS is concerned, Demeestere cited the results of a recent study from her group in 29 patients with early-stage breast cancer tumor showing that the levels of circulating tumor DNA (ctDNA) were not altered after LET-OS [37]. Demeestere also highlighted that a recent meta-analysis encompassing 2121 patients did not find any difference in the number of metaphase II oocytes retrieved in women who had OS with or without letrozole cotreatment [38]. When focusing on women with estrogen receptor (ER) negative breast cancer, Demeestere emphasized that ovarian stimulation may also have a growth-promoting effect via autocrine and paracrine mechanisms. In view of the available data underscoring these mechanisms, she has adopted the LET-OS protocol for all breast cancer patients including those with ER negative disease, although there is currently no clinical evidence to support this approach. In this context, she also presented unpublished prospective data from a center in France showing no difference in breast cancer recurrence rate after a median follow-up of 48 months in 56 patients who had OS without letrozole and 41 patients who had LET-OS, irrespective of the hormone receptor status. Finally, there was no difference in survival rates in more than 400 breast cancer patients in Sweden who had OS with or without letrozole cotreatment [39]. She emphasized the need for studies investigating the effect that factors other than gonadal steroids may have on tumor growth; in this respect, she referred to a study by Radu et al. [40], indicating that endothelial cells at the margin of several types of tumor express increased levels of the follicle stimulating hormone receptor (FSHR), which may promote proliferation and migration of endothelial cells in cancerous tissue, through a pathway independent from VEGF. In view of this and to mitigate the possible effect of OS on tumor growth, she emphasized that it is recommended to start cancer treatment as soon as possible after OS.

Professor Richard Anderson (Edinburgh, UK) reviewed not only the long-term effects of cancer treatment on different aspects in the life of cancer survivors, including health issues and reduced ovarian function, but also social issues including reduced chances for cancer survivors to have a relationship and concerns about starting a family after cancer in view of cancer recurrence risks and altered life expectancy. He cautioned that, while optimized strategies for cryopreservation of gametes or gonadal tissue are key elements of fertility preservation, the ultimate goal of fertility preservation is not the cryostorage itself but a healthy baby after fertility preservation. He highlighted the long history of studies on the effects of chemotherapy on biomarkers of ovarian function and the importance of decades of follow-up to assess the clinical outcomes of fertility and age at menopause. He also urged us to remember the requirement of a healthy uterus to sustain a pregnancy, which is highly relevant in cancer patients who received radiotherapy, especially in young children. Indeed, the age at which a patient had total body irradiation determines her adult uterine function [41]. Anderson went on to present a variety of data from recent follow-up studies and commended the audience to keep in mind that recovery of ovarian function after chemotherapy is highly variable and that recovery is mediated by age, parameters of ovarian reserve, and type of chemotherapeutic agent [42]. He illustrated the use of population-based data to assess the impact of different types of cancer on the chance of subsequent pregnancy. Based on data from Scotland, only 10% of women will achieve a first pregnancy after a diagnosis of breast cancer, which is about half of their age-matched controls [43]. Prof Anderson brought focus to the current status of AMH as a biomarker of ovarian function and the role of AMH in identifying gonadotoxicity and ovarian function recovery in cancer survivors. With reference to data from Hodgkin lymphoma patients treated with ABVD in Scotland [44], he left most in attendance with the view that while pretreatment AMH indeed predicts the likelihood of restoration of AMH levels in these patients, multiple linear regression analysis showed that age rather than pretreatment AMH was an independent predictor of AMH recovery after ABVD. He discussed the data of a multicenter phase 3 randomized controlled trial showing a lower level of gonadotoxicity of a de-escalation scheme of two cycles of BEACOPP, followed by ABVD in advanced Hodgkin lymphoma patients as compared to the regimen where BEACOPP is continued [45], but he also called attention to the observation from that study as well as from other studies that favorable ovarian biomarker levels and preserving fertility were not the same. Indeed, AMH is not a short-term predictor of fertility, but rather a predictor of duration of reproductive live span.

Professor Marie-Madeleine Dolmans illustrated the most recent published data on fertility outcomes after OTT [46]. Endocrine restoration is reached in more than 80% of cases after OTT and persists for over 5 years in more than half of transplanted women. Looking at fertility outcomes, pregnancy and live birth rates are around 40% and 30% respectively with natural conception, and 36% and 21% respectively with IVF after transplantation. Reproductive chances appear to be related to the age at cryopreservation, but not to administration of chemotherapy (CHT) in the months prior to OTC. Indeed, in the series of 285 OTT that was presented, no significant difference is observed in terms of fertility outcomes in subjects with some prior CHT, confirming previously published data in smaller series. These data support the conclusion that OTC is valid and now standard strategy for fertility preservation in women who had already received recent (< 6 months) CHT. On the other hand, data on fertility outcomes after OTT in subjects with previous radiotherapy (RT) show that chances of pregnancy are around 50% in case of low doses of pelvic RT, while they become extremely low (and even zero) in subjects who had received high radiation doses to the pelvis, with a significant damage of both the uterus and the potential pelvic transplantation sites.

Professor Matteo Lambertini (Genova, Italy) reviewed the available evidence regarding pregnancy after breast cancer. According to epidemiological data collected by his group, breast cancer survivors have the lowest likelihood of a subsequent pregnancy [47]. While referring to a recent study by Razeti et al. [48], he addressed the current updated knowledge about the risk of treatment-related premature ovarian insufficiency in breast cancer patients and the options to prevent this. More specifically, he discussed the highly debated use of gonadotropin-releasing hormone agonists (GnRHa) as pharmacoprotective drug during chemotherapy, referring to a recent meta-analysis that confirmed the efficiency of this approach in breast cancer patients [49]. Based on a recent observational, prospective study in a cohort of 223 breast cancer patients in Italy, more than 90% of patients across all age groups accepted to have temporary ovarian suppression with GnRHa, while only 18.2% of patients aged ≤ 40 years accepted to have fertility preservation based on cryopreservation [50]. Lambertini highlighted that this relatively low uptake of fertility preservation strategies in this sample points towards the importance of proper fertility preservation counselling and a need to lifting the barriers that may impede access to fertility preservation strategies. He also warned that failure to address fertility concerns in breast cancer patients may result in early discontinuation and non-adherence to adjuvant hormonal therapy, which are associated with increased mortality according to several studies. Lambertini’s presentation was a plea for raising awareness among breast cancer oncologists with regard to available fertility preservation options and available evidence about safety of pregnancy after breast cancer. Indeed, there is an increasing amount of published data from retrospective studies showing no detrimental impact of pregnancy after breast cancer, irrespective of the hormone receptor status and the germinal BRCA mutation status. He emphasized that there is still uncertainty about the safety of discontinuing adjuvant hormonal therapy in ER positive breast cancer before the standard recommended period of 5 years, to allow women to try to get pregnant. The long-term impact of a 2-year “break” is under scrutiny in a prospective trial investigating the Pregnancy Outcome and Safety of Interrupting Therapy for women with endocrine responsIVE breast cancer (POSITIVE), and the results are awaited. Lambertini went on to review the data of several studies showing that there is no increased risk of congenital abnormalities after pregnancies in breast cancer survivors although these pregnancies are more often associated with increased risk of cesarean section, preterm birth, small-for-gestational age (SGA), and low birth weight (LBW), with the risk of SGA and LBW being highest in women who became pregnant within 1 year after completion of chemotherapy [51]. Finally, he emphasized the importance of the role of the oncofertility team in the follow-up of young cancer patients beyond reproductive concerns, including other aspects of women’s health such as contraception and menopause.

Towards the end of meeting, Prof De Vos (Belgium) presented four clinical cases from the clinic to spark the interest of the attending clinicians and to engage the participants and the panel of expert clinicians in a lively debate on recurrent questions regarding appropriate management of patients in the oncofertility setting. Fuel for debate included the adoption of letrozole cotreatment in breast cancer patients with estrogen receptor negative disease, the choice of contraception in breast cancer patients, and ovarian tissue transplantation in cancer survivors of advancing age because of age-related infertility.
